# Poor Man's Gout

**Published:** 1892-05-28

**Authors:** 


### The Doctor's Armchair.

### POOR MAN'S GOUT.

A correspondent, "Stella," asks the question, "What is meant by 'poor man's gout?'" It is possible that a good many other people beside "Stella" would like an answer to this question, and we therefore give here such a reply as may help, in some measure, to solve the difficulty.

To begin with, we should be inclined to say, speaking broadly, that there is no such thing as "poor man's gout." Gout is gout all the world over, whether it occur in the poor or in the rich. Perhaps it may help a little if we strive to give some idea in simple language of what gout actually is. In thinking of this, as of other diseases, it is best to associate it with a particular individual, and instead of trying to reason about gout to reason about a gouty person. Let a gouty man be our concrete study. The writer has one such in his mind's eye, whom he examined two or three days ago. He was a landed proprietor, of about forty years of age. In build he was square and solid, broad-shouldered, deep-chested, muscular, with a full sized head, set firmly on a neck rather short and massive like a Norman tower. Altogether he was a man of mighty proportions. He looked robust and healthy in a very high degree. He was a gentleman of temperate habits, and always had been so: from boyhood he had been moderate in food and drink; he had also lived much out of doors, and had accustomed himself to a great deal of riding, walking, shooting, and other open-air exercise. And yet this man of forty had had numerous attacks of gout. In boyhood, almost in childhood, he had made the acquaintance of his enemy; and on all manner of occasions since that time he had been the subject of periodical attacks.

Now this gentleman was the subject of gout, and of gout which might, in one sense, be called the "poor man's" variety; that is to say, he had been born with such a gouty constitution that, whether poor or rich, self-indulgent or abstemious, he would all his life have been subject to gouty seizures on the smallest possible provocation.

What was there in the man's body, in his flesh or in his blood, or in his lymphatics, in his brain or in his bones, in his liver, his kidneys, his spleen or his lungs, to make him the subject of repeated attacks of gout? There was in some or all, most probably all, of his organs and tissues a tendency to the production of uric acid in excess; or there was a deficiency in the quantity of alkalies in his blood to combine with the uric acid and carry it off; or there was an impairment of excreting power in the organs of elimination. It is obvious that all we can do here is to state these facts and hypotheses in the baldest way. There is no space for their proper discussion, nor would such a discussion be profitable for our present purpose.

Bearing in mind, however, that the most striking characteristic in a gouty person, from the physiologist's point of view, is the presence of an excess of uric acid in his blood and tissues, we may be able by a little reasoning to give "Stella" and other readers some principles whereby they may decide for themselves what kind of gout it would be proper to call "poor man's gout." Two circumstances are here to be noted: first that gout is often hereditary, and secondly that gout may be acquired. It is obvious that a poor man may as easily inherit gout as a rich one. The poor man may have had a rich father, or a rich mother. But if he be poor himself, and if he has always been poorly fed and cared for, and has never indulged in either meat or drink to excess, and yet has gout, what sort of gout can it be but "poor man's gout"? If we remember that gout is always gout and never anything else, it is plain that the only distinction between poor man's gout and rich man's must be in the men and not in the gout. Of course, it must be understood, that a man may be poor in body, poor in blood, poor in strength, without being poor in pocket. "Poor man's gout" is that gout which abides in and afflicts a man who is physiologically poor, whether he be poor in purse or not.

Thus far we have considered the question of hereditary gout, and we have endeavoured to show that a poor man may inherit gout as well as a rich one, although, of course, the children of rich parents are much more likely to inherit gout than the children of poor ones. In considering the second variety, viz., "acquired gout," it may not unnaturally be supposed that poor people are very unlikely to acquire the disease by their mode of living. But this would be a decided mistake. Even among the poor there are certain causes that operate to set up gout of the variety which may properly be called "acquired." Here it is desirable to point out that just as a rich man may have poor man's gout, so a comparatively poor man may have rich man's gout. For, as we have already stated, it is physiological and not finanical poverty that determines the quality of the seizure.

Acquired gout is usually one of the consequences of errors and excesses of diet. Those who eat too much meat and drink too much wine are, as is well-known, very frequently the subjects of the disease. But it is by no means so well-known that beer is a prolific cause of gout. The fact is so, however. Dr. Frederick Roberts in *Quain's Dictionary of Medicine*, tells us that brewers' draymen are particularly subject to gout. Malt liquors, Dr. Roberts considers, stand next to wines as originators of gout. Good whiskey and brandy on the other hand are said to be much less mischievous in this connection. Brewers' draymen, though comparatively poor in pocket, do not generally suffer from poor man's gout. On the contrary, their gross and ponderous bodies are gorged with the products of their own excesses. Some of them, it is said, drink as much as two to four gallons of beer a day. Sir Alfred Garrod, a competent authority, states that lead taken into the system is a potent cause of gout. No less than thirty per cent. of Dr. Garrod's hospital patients owed their gouty seizures to working among lead. Many of these were probably physiologically poor, poor in blood and tissue; and they would, no doubt, suffer from what is popularly called poor man's gout. Butchers and barmen, coalheavers and painters, and others who have to do with lead are all liable to the disease.

It will be proper here to remind readers that mental worry is a fruitful source of gout; and as the mentally worried often have poor appetites and feeble digestive powers, they are not seldom physiologically poor in a very marked degree, and they are, therefore, peculiarly liable to suffer from poor man's gout. Scientifically, this question of terms and definitions has little or no interest, because, as we have already said, gout is gout, and can never be anything else. But from a practical point of view the subject is well worthy of the consideration of all intelligent persons.

